# Multistable network dynamics through lateral inhibition: an efficient mechanism for selective information routing

**DOI:** 10.1186/1471-2202-15-S1-P165

**Published:** 2014-07-21

**Authors:** Daniel Harnack, Klaus R Pawelzik, Udo A Ernst

**Affiliations:** 1 Institut für Theoretische Physik, Universität Bremen, Bremen, Germany

## 

A remarkable feature of the brain is its ability to flexibly process information in a context-dependent manner. A hallmark example is selective visual attention. It allows to focus on relevant information, and to ignore distracting features of a visual scene, effectively routing information through the visual hierarchy [1].

A seminal hypothesis about how this routing is accomplished is 'Communication through Coherence' (CTC) [2]: A neuron with oscillating membrane potential can be driven to fire by a relatively weak input when it is close to the threshold, while if it is in a phase of low excitability, even stronger input might fail to elicit an action potential. Thus, shifting relative phases of oscillating sending and receiving populations could be utilized to gate information.

Here, we investigate neural mechanisms of information routing and their effectiveness in a generic, two-layered cortical network model with lateral recurrent interactions.

Building on the CTC hypothesis, we construct a biophysically plausible network model mimicking the fan-in structure of the visual processing stream. It comprises four populations A, B, C and D of recurrently coupled networks of conductance-based spiking neurons. A and B correspond to sending populations in a lower visual area and have non-overlapping receptive fields. They project to receiving populations C and D in a higher visual area with larger receptive fields. Upon activation, all populations engage in oscillatory activity.

We show that introducing distance-dependent lateral inhibition brings the receiving populations in phase, and the sending populations in anti-phase, thus establishing a phase competition between the two sending populations: Only one population can be in a favorable in-phase relation with the receiving populations, since the other is automatically forced into anti-phase, thus hindering information transfer. This 'self-organization' of phase relations mediated by lateral inhibition distinguishes our approach from previous modeling efforts (e.g. [3-5]).

When increasing the strength of the lateral inhibition between A and B, the system transitions from a regime of mixed representations to a bistable one, where one sending population is in a favorable in-phase relation for an extended period of time. In this regime, a brief current pulse to one sending population is sufficient to set a desired phase relation and thus 'switch' information routing in the network. This mechanism bears two interesting consequences: First, it allows neural circuits to control routing using energy-efficient, short bursts of neural activity instead of requiring sustained elevation of firing rates. Second, such pulses delivered by electrical stimulation could be employed to externally direct the focus of attention, a possible application in the field of brain prosthetics.
